# 4086 HIV-Associated Myocardial Diastolic Dysfunction and Soluble ST2 Concentration in Tanzanian Adults: A Cross-Sectional Study

**DOI:** 10.1017/cts.2020.300

**Published:** 2020-07-29

**Authors:** Justin Kingery, Rahul Hosalli, Bernard Desdarius, Fredrick Kalokola, Edmund Damas, Karl Reis, Hyung Hee Lee, Margaret McNairy, Daniel Fitzgerald, Robert Peck

**Affiliations:** 1 Weill Cornell Medicine; 2 Weill Bugando School of Medicine

## Abstract

OBJECTIVES/GOALS: To determine the prevalence of myocardial diastolic dysfunction (DD) and association of serum concentration of the cardiac biomarker serum soluble ST2 in HIV-infected as compared to uninfected Tanzanian adults at the time of HIV diagnosis. METHODS/STUDY POPULATION: In this cross-sectional study we consecutively enrolled HIV-infected participants and uninfected controls at a large, referral HIV clinic in Mwanza, Tanzania. Standardized history, physical examination, echocardiography and serum samples were obtained. The primary outcome was prevalence of myocardial diastolic dysfunction in HIV-infected as compared to uninfected adults. The secondary outcome was the association of baseline serum sST2 concentration with diastolic dysfunction prevalence. Regression models were used to quantify the associations. RESULTS/ANTICIPATED RESULTS: We enrolled 388 HIV-infected, ART naïve and 461 HIV-uninfected controls. Participants with HIV had a higher prevalence of DD (OR = 2.44, p = 0.001, controlled for age, sex, hypertension and BMI) and more severe dysfunction (66.7% vs 42.5%, p = 0.056) at an earlier age. Baseline serum sST2 concentration was significantly associated with DD in HIV-infected but not uninfected participants (p = 0.04 and 0.90, respectively). More HIV-infected adults with concurrent DD exceeded the threshold of 35ng/mL as compared to controls (15.7% vs 5.3%, p<0.0001). Additionally, a significant population level shift to higher sST2 concentration was observed in HIV-infected adults with dysfunction as compared to both HIV-infected without and HIV-uninfected adults with dysfunction (Kolmogrov-Smirnov test: p = 0.02 and 0.04). DISCUSSION/SIGNIFICANCE OF IMPACT: In a large population of HIV-infected adults in sub-Saharan Africa, HIV infection is associated with myocardial diastolic dysfunction. This dysfunction is associated with higher sST2 concentrations. Therefore, we conclude that the sST2 pathway may provide insight into the pathophysiologic mechanisms of dysfunction in HIV-infected adults.

